# DTI tractography of lissencephaly caused by TUBA1A mutation

**DOI:** 10.1007/s10072-014-1662-3

**Published:** 2014-02-08

**Authors:** Kouhei Kamiya, Fumine Tanaka, Mitsuru Ikeno, Akihisa Okumura, Shigeki Aoki

**Affiliations:** 1 Department of Radiology, Juntendo University Graduate School of Medicine, 2-1-1, Hongo, Bunkyo-ku, Tokyo, 113-8421 Japan; 2 Department of Pediatrics, Juntendo University Graduate School of Medicine, 2-1-1, Hongo, Bunkyo-ku, Tokyo, 113-8421 Japan

Several genetic causes have been identified in lissencephaly, including mutations in LIS1, DCX, ARX, RELN, VLDLR, WDR62, and TUBA1A genes. One may sometimes suggest the most likely gene mutation upon MRI findings when some specific features are seen, although image findings overlap among the causative gene mutations.

**Fig. 1:**
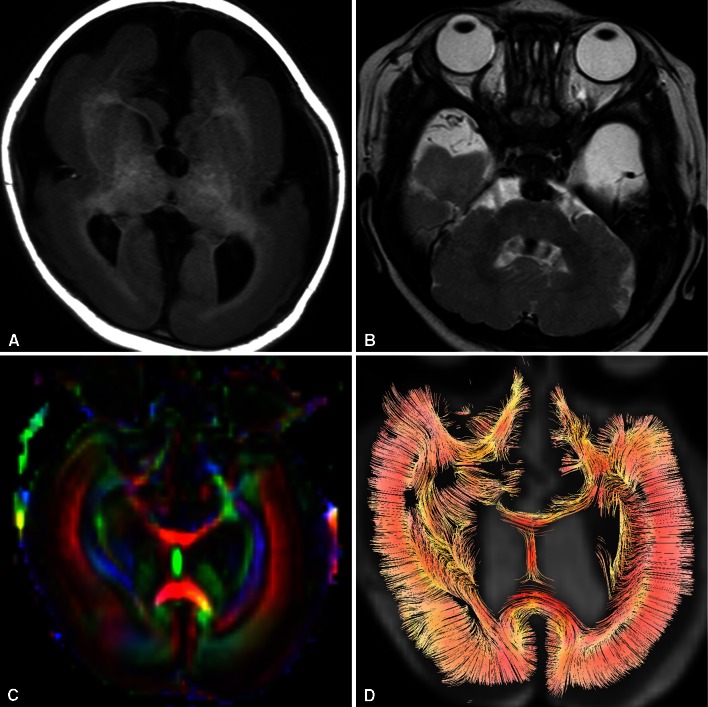
T1-weighted axial image (**a**) and T2-weighted axial images (**b**) show diffuse thickening of the cortex with lack of sulcation, accompanied by severe dysplasia of the brainstem and cerebellum. Obscuration of the contour of the basal ganglia and internal capsule is also demonstrated. FA colormap (**c**) and tractography (**d**) reveal radially oriented anisotropic structure within the thickened cortex, which is not normally observed after birth

A 1-year-old boy presented with intractable epilepsy, developmental delay and microcephalus. Brain MRI revealed lissencephaly with severe dysplasia of the cerebellum and brainstem. Diffusion tensor imaging (DTI) visualized radially aligned structures within the cortex, which is not observed in normal brain after birth (Fig. 1). Combination of severe posterior fossa abnormalities with lissencephaly suggested TUBA1A as the most likely responsible gene mutation. Mutation analysis of TUBA1A gene revealed a missense mutation of c.1205G>A, which was negative in both parents indicating a de novo mutation.

Mutation of TUBA1A gene, coding for alpha I tubulin that constitutes subunit of microtubule, is a relatively rare cause of lissencephaly. The most consistent feature of lissencephaly due to TUBA1A mutation is severe cerebellar and brainstem abnormalities. Other characteristics include dysmorphic basal ganglia with hypoplastic internal capsule, callosal hypoplasia, and microcephaly, as seen in this case [1, 2]. Recently, mutations in the neuronally expressed tubulin genes have been reported to cause a spectrum of overlapping cortical malformations ranging from lissencephaly to polymicrogyria, and categorized as “tubulinopathy” [3]. Although cerebellar abnormalities may be present in LIS1 and DCX mutation, they are much less severe than in TUBA1A mutation.

The radially aligned structure within the cortex and increase of cortical fractional anisotropy (FA) are transiently observed in the normal developing brain around 15–28 gestational weeks [4]. Normally, the cortical FA declines with development and approaches zero by 36 weeks [5]. This transient radially aligned anisotropic structure has been presumed to represent the presence of radial glia [4, 6]. The radial glias retract their ventricular and pial attachments and differentiate into astrocytes as the brain matures normally, with corresponding decrease in the cortical FA value. The radially oriented structure within the cortex demonstrated in this case may represent remains of radial glia or persistent radial orientation of neurons arrested in the migration process [7].
